# Workplace health in anesthesia team: A qualitative study in Iran

**DOI:** 10.3389/fpubh.2023.1141447

**Published:** 2023-03-03

**Authors:** Ali Khalafi, Nooshin Sarvi Sarmeydani, Sara Adarvishi

**Affiliations:** ^1^Department of Anesthesiology, School of Allied Medical Sciences, Ahvaz Jundishapur University of Medical Sciences, Ahvaz, Iran; ^2^Department of Nursing, School of Nursing and Midwifery, Ahvaz Jundishapur University of Medical Sciences, Ahvaz, Iran

**Keywords:** nurse anesthetist, anesthesia team, workplace health, occupational, health nursing, anesthesiologist

## Abstract

**Background:**

All anesthesia providers, including nurse anesthetists and anesthesiologists work in a stressful environment with diverse tasks. The profession is characterized by high workload, both dependent and independent job descriptions, and unpredictable conditions. This study was designed and conducted to explain the factors affecting the workplace health of Iranian anesthesia teams.

**Methods:**

Twenty anesthesia team members including nurse anesthetists and anesthesiologists from 7 different hospitals were enrolled in this phenomenological research. The data were collected in 2022. Semi-structured interviews were used for data collection, and the transcripts were analyzed using qualitative content analysis.

**Findings:**

The most notable theme emerging from the data which was found to affect workplace health was consistency of anesthesia team. Other themes derived from the data included team tranquility and physical well-being.

**Conclusion:**

The participants' emphasis was more on behavioral and managerial factors affecting workplace health, and desirable interpersonal cooperation to create a suitable work environment for them was more prominent. These findings can raise the awareness of chief nurse anesthetists and planners to provide more effective teamwork, modify the job description structure, and reduce staff conflicts.

## 1. Introduction

All anesthesia providers, including nurse anesthetists and anesthesiologists work in a stressful environment with diverse tasks. The profession is characterized by high workload, both dependent and independent job descriptions, and unpredictable conditions (1). Studies show that long-term stress has significant physical and psychological consequences for health care workers, which can affect their health and quality of life, and may even affect patient care (2, 3). Working in a stressful environment can lead to an unbalanced life (4, 5).

Based on WHO's definition of workplace health, there is an emphasis on the cooperation of employees and managers to create a process of continuous workplace improvement and promotion of health, safety and stability. Also, “Physical work environment”, “Psychosocial work environment”, “Personal health resources” and “Enterprise community involvement” are seriously affected in the anesthesia work environment (6).

In Iran like most other countries, there are two different anesthesia care providers, namely nurse anesthetists and anesthesiologists. A nurse anesthetist has a bachelor's degree in anesthesia. They take care of patients' anesthesia needs before, during, and after surgery with the assistance, advice and supervision of anesthesiologists. When anesthesiologists and nurse anesthetists work together, the nature of their interactions can affect their patient care (7, 8). Due to their many overlapping skills, when it comes to allocation of work tasks, nurse anesthetists and anesthesiologists may have conflicts. However, high efficiency and a sense of well-being could be promoted among team members by a good team climate (9). Staff motivation can be affected by differences between members of the anesthesia team. Also, individual, interpersonal and organizational factors, as well as conflicts, unequal power relations and mistrust among anesthesia staff can affect how they respond to everyday conflicts (10). Effective workplace relationships are essential for a healthy workplace (11). Nurses want a desirable interdisciplinary relationship since an effective nurse-physician relationship is one of the hallmarks of a satisfying and productive work environment (12, 13). This mutual relationship has been described as the basis of mutual trust, power and respect between the parties in a workplace (14). Chief nurse anesthetists have a special responsibility to prepare the grounds for team members to interact with each other, and as a result patient safety and outcomes could be optimized (15, 16). The relationship between nurse and chief nurse anesthetist and peer relationships are very important for a healthy workplace. The role of the chief nurse anesthetist determines the work environment and affects its dynamics (17, 18). Various studies have reported significant levels of incivility and aggression in the anesthesia team workplace (19, 20). Aggression in the workplace is a serious problem that is on the rise and is a major concern because of the wide range of consequences including a negative work environment and reduced employee well-being. Behaviors that contribute to aggression in the workplace include backstabbing, negative criticism, lack of support, unwillingness to help, social deprivation, and isolation (20). All of these factors seriously upset the work environment and create an unhealthy atmosphere. Nurse anesthetists work in a unique environment and have responsibilities beyond the scope of nursing, which places them in a unique position, separate from others in the nursing profession. Working in an unhealthy workplace is expected to increase the likelihood of burnout among these health professionals (21). This situation is more serious for nurse anesthetists in Iran because they do not have a general nursing background and enter directly into the four-year program of anesthesia nursing to receive a bachelor's degree. This seems to be the root of most of their problems and conflicts at workplace and their greater differences with general nurses. Therefore, long working hours, stressful workplace environment, and nurse-physician relationship can negatively affect the health of nurse anesthetists at work, due to psychosocial stressors and cultural factors. To the best of our knowledge, no study has examined the factors affecting the specific work environment of anesthesia teams in Iran. Unlike most other countries, nurse anesthetists in Iran are relatively independent of the general nursing staff in terms of both their education and organizational affiliation, and the lack of disciplinary and managerial support for them is evident. Therefore, the present study was conducted to explain the factors affecting the health of the workplace of anesthesia teams in Iran. To this aim, the current research lends itself to provide answers for the following research questions:

1. What is the nurse anesthetist and anesthesiologists' perspectives on health of the workplace in anesthesia environment?
2. What factors (Including individual, interpersonal, managerial, and organizational) affect the health of work environment of nurse anesthetists and anesthesiologists?

## 2. Methods

A phenomenology design was employed in the study to deepen our understanding of a healthy work environment in a natural setting. Personal interviews were conducted, and qualitative content analysis was performed. By careful examination and constant comparison, qualitative content analysis delves into the depth of individuals' experiences of specific phenomena. Its primary goal is to provide knowledge and understanding of the phenomena under study.

### 2.1. Setting

The participants of this study were selected from among all anesthesia care teams who worked in seven different hospital anesthesia departments in Ahvaz. All 7 hospitals were affiliated with Ahvaz Jundishapur University of Medical Sciences (AJUMS) and they were in almost the same in terms of environmental, managerial, and organizational conditions including employee salaries and benefits. The participants were eligible to participate in the study if they: were anesthesiologists or nurse anesthetists with more than 2 years of clinical experience and had the ability to speak Persian to participate in the interviews. The exclusion criteria was having speech disorder or prior relationship with the research team. The number of staff in each department ranged from 15 to 50. Initial contact with the potential participants was made by written invitations placed in boards of the anesthesia departments in operating rooms explaining the study objectives and asking them to contact the lead researcher *via* telephone if interested in participating. Thirty-eight employees (32 nurse anesthetists and 6 anesthesiologists) responded of whom 20 were chosen using a purposive sampling method allowing maximum variation in terms of age, gender, and experience to reflect the genuine structure of the anesthesia team in Iran. Sampling continued until data saturation (10). To achieve maximum diversity in terms of the participants interviewed, data collection was initiated with nurse anesthetists and then extended to the anesthesiologists.

### 2.2. Ethical considerations

This study was approved by the Ethics Committee of Ahvaz Jundishapur University of Medical Sciences (Ref. ID: IR.AJUMS.REC.1399.702), and an introductory letter was given to the lead researcher to conduct the study in the designated centers. Before starting the study, the lead researcher introduced himself to the participants, and they were briefed on the objectives of the study, the voluntary nature of participation in the study, withdrawal from the study at any time, the reason behind voice recording, information confidentiality, and the accessibility of information for all participants. Finally, informed consent was obtained from all participants.

### 2.3. Data collection

Qualitative, intensive, in-depth and semi-structured interviews were used to collect the required data. The interview guide included a number of open questions to allow participants to express their perceptions and experiences in detail. At the beginning of each interview, the participants were asked to discuss their experience of health in relation to their work environment. They were then asked to describe their perceptions and experiences about the factors (including individual, interpersonal, managerial, and organizational) that affect the health of anesthesia teams' work environment. Focusing on the anesthesia team structure, the main questions were asked to extract ways to contribute to a better health-promoting work environment. The participants were also asked to give clear and tangible examples of their experiences. The transcript of the audiotaped interviews was written on the same day by the first author and was used as the original data. Data collection was conducted over 6 months from May 2022 to November 2022. Each interview was conducted in one session in a private room at the operating rooms unit after the working shift. Each session lasted from 40 to 90 min. To prevent the negative impact of participants' exhaustion on the interview, the interviews were conducted at the end of a shift when the workload of the participant was low or when the operating room was not active.

### 2.4. Data analysis

Data were analyzed through content analysis following Lewis and Malecha (22). Each step of the content analysis was first performed by one person (the interviewer) and then discussed and checked with colleagues in the next stage. This method of analysis includes the following steps:

(1) The content of the recorded interviews was transcribed verbatim and reviewed by the researchers several times to reach a general understanding.
(2) Words, sentences, or paragraphs that were linked in terms of content were regarded as semantic units. The semantic units were summarized based on their content.
(3) The semantic units reached a level of abstraction and conceptualization and were labeled by codes according to the concept underlying them.
(4) The codes were compared according to their similarities and differences and classified with specified labels in more abstract classes.
(5) Finally, by comparing different classes with deep and careful reflections, data content was introduced as the theme of the study.

### 2.5. Rigor of the study

The Lincoln and Guba (23) criteria were used to measure the rigor of the study (23). Strategies were adopted to ensure the credibility of the data: tape-recording and verbatim typing of transcripts, prolonged engagement of the researcher in the study (May to November), member check by participants to approve the interpretations of the researchers, and checking the preliminary categories by an expert chief nurse anesthetist and two faculty members of anesthesiology department. As far as dependability was concerned, coding of the interviews was carried out again by another co-author who had experience in coding qualitative data. Moreover, the researchers documented the research details to allow for the possibility of external review.

## 3. Findings

A total of 20 participants including 14 nurse anesthetists, 3 chief nurse anesthetists and 3 anesthesiologists from 7 different hospitals were interviewed. Of the 20 participants enrolled in this study, 12 were females and 8 were males. Table 1 shows the demographic data of the participants.

**Table 1:** Demographic characteristics of participants.

**Demographic characteristics of participants**	**Nurse anesthetist *n =* 14**	**Chief nurse anesthetist *n =* 3**	**Anesthesiologist *n =* 3**
**Age**			
21–30	4	0	0
31–40	5	0	1
41–50	5	2	1
50–60	0	1	1
**Gender**			
F	9	2	1
M	5	1	2
**Experience in OR**			
<5 years	4	0	0
6–10 years	5	1	1
>10 years	5	2	2

From the deep and rich descriptions provided by the participants, 1,084 semantic units were extracted. After several reviews, the semantic units were summarized and classified on the basis of similarity. They were then summarized once again into three main themes and eight conceptual and abstract subthemes. The three main themes included consistency of the anesthesia team, team tranquility, and physical well-being (Table 2).

**Table 2:** Study themes.

**Theme**	**Sub-themes**
Consistency of anesthesia team	• Perceptive and adaptive management • Stability in team composition • Adherence to the defined job description
Team tranquility	• Coworker trust • Avoiding conflicts • Interpersonal justice
Physical well-being	• Operating room structure • Workplace accommodations

### 3.1. Consistency of anesthesia team

Participants considered the consistency of anesthesia teams as one of the most important strategies to promote workplace health. The anesthesia team operates in a stressful and unpredictable environment that is influenced by various individual, organizational and managerial factors. Lack of stability and acting based on personal taste on the part of both anesthesiologists and nurse anesthetists will negatively affect the results of their actions and lead to conflicts at workplace. Participants identified three key strategies effective in consistency the anesthesia team, namely perceptive and adaptive management, stability in team composition, and adherence to the defined job description.

#### 3.1.1. Perceptive and adaptive management

The main emphasis of most participants was on the management of anesthesia staff. In Iran, every operating room has a chief nurse anesthetist who is responsible for all activities of anesthesia nurses, including allocation of staff into operating rooms, coordination of the anesthesia team, and resolving possible problems and conflicts. Chief nurse anesthetists are on a regular morning shift every day and monitor staff performance. Arranging anesthesia teams in operating rooms according to the type of surgical procedure and assigning anesthesia nurses to the teams of each anesthesiologist are among their important responsibilities.

“*The work of the chief nurse anesthetist is very influential in the structure of our team. It is important how tactfully he distributes the staff in the operating rooms. If the chief nurse anesthetist knows the staff well and knows the capabilities and personality traits of the staff, the most successful teams could be formed.”* (Nurse anesthetist No 3)

“*Experienced and knowledgeable chief nurse anesthetists prevent a lot of conflicts and frustration among staff by properly dividing the workforce.”* (Nurse anesthetist No 7)

From the participants' point of view, it was important for chief nurse anesthetists to pay close attention to the details of verbal and non-verbal behaviors of the staff as well as their motivations and abilities, and to have timely intervention when problems arise in promoting workplace health. In order to increase nurse anesthetists' satisfaction with their job, chief nurse anesthetists need to meet their physical and mental needs and establish balance between the newcomers and the experienced staff.

“*Chief nurse anesthetists should not only focus on their routine tasks, but also be meticulous and able to identify the needs of their employees.”* (Chief nurse anesthetist No 2)

#### 3.1.2. Stability in team composition

In this study, the participants were selected from 4 university hospitals and 3 non-university hospitals. In university centers, although nurse anesthetists are fixed members of the anesthesia team, the presence of anesthesia residents as well as anesthesia nursing students in the operating rooms causes frequent changes in the composition of the anesthesia teams. In non-university centers, however, both anesthesiologists and nurse anesthetists are fixed members and are only distributed in different work shifts, leading to more stability in the composition of the members of the teams. This difference allowed the researcher to examine the frequent changes in the composition of the anesthesia team from the participants' point of view. The participants pointed out that when working with the same people in the team for a long period of time, they gain a good understanding of each other's level of theoretical knowledge, practical skills, and personality traits. This leads to an improved communication and greater coordination in the performance of the anesthesia team, making the staff feel less stress.

“*Mutual recognition and trust come from constant collaboration. I have been working with nurse anesthetists here for almost 5 years. We know what to expect from each other and we have a stress-free environment.”* (Anesthesiologist No 1)

#### 3.1.3. Adherence to the defined job description

A common problem for members of the anesthesia team is the overlap between the duties of anesthesiologists and nurse anesthetists. According to the participants, the following were the common problems associated with job description: lack of any precise definition of job descriptions, ineffective briefing of employees on their job description, lack of adequate supervision over proper implementation of job description, and as a result, lack of commitment of most anesthesia staff to their performance. Doing things that are outside the scope of specific job descriptions often leads to neglecting certain tasks, and under such circumstances, everyone waits for someone else to take the initiative and do that task. In addition, other consequences of undefined job description, from the participants' point of view, were conflicts between members of the anesthesia team, which will ultimately lead to increased stress.

“*Here we do not know exactly what is considered as our duty and what counts as the duty of anesthesiologists. We have to adapt ourselves with different anesthesiologists whose expectations are different. This causes confusion and boredom.”* (Nurse anesthetist No 6)

### 3.2. Team tranquility

The second theme derived from the participants' experiences was Team tranquility. According to the participants, a calm and less stressful environment is a basic condition for the optimal performance of the team and creating a healthy atmosphere in the workplace. They pointed to three important strategies for achieving such an atmosphere: coworker trust, avoiding conflicts, and interpersonal justice.

#### 3.2.1. Coworker trust

Anesthesia teams are formed by a limited number of members, often including only a nurse and an anesthesiologist. At university centers, anesthesia residents and anesthesia nursing students may be added to these. The team members work together frequently and continuously, and by increasing their knowledge of each other's capabilities and expectations, a feeling of mutual trust is created between them. From the participants' point of view, this trust provides comfort while providing care at the patient's bedside. Of course, there is a degree of distrust when it comes to working with newcomers. Also, a comparison between the experiences of university staff (where there is frequent changes in the clinical rotations of residents and students) and the private centers (where the team structure is almost fixed) shows the formation of more trust between team members in the latter.

“*There are far fewer problems when staff work together for long periods of time. They know each other's working style better and can trust each other in certain situations.”* (Chief nurse anesthetist No 1)

“*Here we have both a resident and a nursing student. There isn't such a thing as a fixed anesthesia team, and members change every day. This does not allow for the establishment of appropriate work relationships and mutual trust.”* (Anesthesiologist No 3)

#### 3.2.2. Avoiding conflicts

Due to the dense and busy nature of the anesthesia work environment, interpersonal and interdisciplinary conflicts are commonplace. According to the participants, the way staff cope with conflicts is different. However, conflict avoidance was introduced as a more successful solution than conflict resolution. They believed that in a situation where the staff are less likely to get involved in an atmosphere of controversy and competition, conflicts along with subsequent discomfort and stress are less likely to occur. The more intra- and inter-team conflicts are controlled, the more tranquility will be created in the anesthesia team.

“*Experience has taught me that I should not argue with my co-workers or the anesthesiologists. I'd better get over some issues. That way I feel more relaxed. This is because I am the one to be harmed in conflicts.”* (Nurse anesthetist No 11)

*Employees are responsible for keeping their work environment healthy. Some are always looking for competition and tension and behave inflexibly. These conflicts make things difficult for all of us.”* (Nurse anesthetist No 5)

#### 3.2.3. Interpersonal justice

Members of the anesthesia team noted fairness and being fair in behavior as important factors contributing to interpersonal justice. Justice-based behavior was sought by chief nurse anesthetists, colleagues, and the organization. The participants believed that chief nurse anesthetists' observance of equality in patient allocation and distribution of personnel to different shifts and their fair division of tasks and facilities are effective on the well-being of the anesthesia team members.

“*I try to be as fair as possible in allocating nurse anesthetists into operating rooms. If we always leave longer and more difficult surgeries to certain employees, they will be under a lot of pressure. They would compare themselves to their colleagues and this would disturb their peace and lessen their motivation.”* (Chief nurse anesthetist No 2)

Our findings showed that another aspect of justice lies in the treatment of anesthesiologists with nurses. They may exhibit different behaviors depending on the level of experience, their intimacy with the nurse anesthetists, or the mental background they have of these nurses.

“*Some anesthesiologists do not treat us all the same. They are too hard on novice nurses and give them less practical help. I can't make sense of this inequality and it upsets me.”* (Nurse anesthetist No 12)

Also among the statements of the participants there were hints of dissatisfaction with organizational inequality between them and other nurses. They described the anesthesia workplace as too crowded, busy, and stressful compared to other wards, and believed that they were not appreciated for their efforts.

### 3.3. Physical well-being

Physical well-being, defined as the feeling of comfort and physical security in the operating room space, was considered as an effective factor in the health of the workplace. According to the participants, the two main aspects of physical health were operating room structure and workplace accommodations.

#### 3.3.1. Operating room structure

The experiences of the anesthesia team members showed that the structure of the operating room has a significant role in facilitating their activities and controlling their fatigue and exhaustion. For example, proximity of operating rooms to the nursing station and the lounge can reduce frequent staff walking. In their view, sufficient operating room space for anesthesia staff to move around the patient's bed without disturbance, and the availability of anesthesia equipment and drugs, lead to less energy expenditure and more focus on patient care.

“*Architectural design standards must be carefully observed in the structure of the operating room. For example, in some centers, the distance between operating rooms is too great, and on busy days I have to cover two or three rooms at the same time. Frequent walking this route is very tedious and time consuming.”* (Anesthesiologist No 2)

Others insisted on providing ample space for rest, meetings, and anesthesia counseling. Due to the restricted space of the operating room and the constant presence of the anesthesia staff during an 8-h shift, the participants noted the importance of effective air conditioning and adequate lighting in reducing fatigue and stress.

“*Our workplace has limited space with a lot of equipment and staff. What's more, there is no opportunity to leave the ward to relieve fatigue and take a breather. It will be very difficult to bear.”* (Nurse anesthetist No 8)

#### 3.3.2. Workplace accommodations

Workplace accommodations were identified as any the availability of any equipment which contributes to the well-being of anesthesia staff in the operating room. Long hours of standing on the patient's bedside leads to fatigue and eventually getting bored with the work environment. The participants insisted that they needed to rest and relax between surgeries. This was said to be achieved by providing facilities such as a roomy lounge equipped with facilities for eating and drinking. Also, the use of ergonomic chairs for long sitting on the patient's bedside was a major factor in promoting physical health from the perspective of the interviewed anesthesia staff.

“*Colleagues in each shift find short opportunities between operations to rest, which must be a comfortable room with sufficient facilities. A room with a bed and facilities for serving drinks and watching TV in those few short minutes; this helps a lot to refresh the staff*.” (Chief nurse anesthetist No 1)

## 4. Discussion

The most important finding of the present study was the emphasis on maintaining team unity in the promotion of health in the workplace. As nurse anesthetists and anesthesiologists work together to provide anesthesia care, a platform for close and ongoing interdisciplinary collaboration is created. Most of the health threats involve the entire anesthesia team, and health promotion strategies should be sought in the management of the anesthesia team.

Consistency of the anesthesia team as one of the main themes of this study means formation of stable anesthesia teams and establishment of interpersonal relationships between their members. While nurse anesthetists are independent professionals, their performance depends largely on the competence, skills and willingness of anesthesiologists to cooperate with them to provide optimal care during anesthesia (24). To date, most attempts to understand the complex nature of the physician-nurse anesthetist collaboration have been subjective and speculative (25). The nurse anesthetists' level of dependence on anesthesiologists and their expectations from them vary depending on their personality, ability, and experience. This leads to inconsistencies and conflicts, which are ultimately followed by stress and unhappiness in the team. Nurses anesthetists and anesthesiologists have many overlapping skills, so assignment of tasks can be a source of conflict (21). However, the team atmosphere can increase efficiency and a sense of well-being in team members (15).

In this study the effective techniques of chief nurse anesthetists in controlling this condition were defined as perceptive and adaptive management. The managerial capabilities of chief nurse anesthetists in a complex and dense anesthesia work environment play a vital role in improving the health of team members. Chief nurse anesthetists' purposeful planning and tactful organization of forces to form anesthesia teams leads to stability in team composition. The distribution of forces and patient allocation should be based on not only their level of knowledge, ability, and experience but also the surgical procedure and anesthesia methods used. This helps to form strong anesthesia teams with the least change in composition. Chief nurse anesthetists have a special responsibility to create opportunities for team members to communicate with each other and thereby enhance patient safety and outcome (26). By the same token, Dexter and Franklin introduced management as the organizer of the situation and emphasized the role of chief nurse anesthetists in creating a healthy and supportive work environment, from both collaborative and health-promoting perspectives (27). Adherence to the defined job description in this study indicated the unclear and inconsistent demarcation of the duties of nurses and anesthesiologists. In other words, while the anesthesiologist is responsible for the technical and medical treatment, the nurse anesthetist has to take care of the patient's general safety. However, in practice, many tasks that are legally within the range of responsibilities of anesthesiologists are performed by anesthesia nurses. This gives rise to a hidden competition in the members of the anesthesia team that can distort the identity and independence of each member (28).

Another theme of the study was Team tranquility, which generally emphasized an atmosphere of trust, justice, and collaboration in the operating room among anesthesia colleagues. Coworker trust and avoiding conflicts are directly related and largely indicative of the same thing, namely team tranquility. Interpersonal trust is built upon members' knowledge of each other's expectations as well as ensuring each other's professional skills and competence (29). Anesthesiologists generally rely more on nurse anesthetists who are aware of their skills, and this gives the overall peace of mind. From the participants' point of view, this is significantly effective in reducing conflicts in anesthesia teams (30). In this regard, Hancock et al. have reported negative team dynamics including poor communication, lack of trust and respect, and violence in the ICU as a factor for burnout of nurses and doctors (31). Another part of the concept of avoiding conflicts depends on the personality of the anesthesia staff. People who are inherently forgiving and patient work more easily in anesthesia teams (32). Interpersonal justice as a complement to the other two sub-themes was further emphasized by the participants as the responsibility of the nurse anesthetists in any ward. Chief nurse anesthetists can create a healthier atmosphere among their staff by observing justice while assigning tasks to the staff. This includes taking into account the number of patients, the type of surgery, and the shift plan, which will prevent many conflicts in anesthesia teams (27). Almodibeg et al. also found that incompetent managers with unfair and unsupportive behavior are considered sources of workplace stress for nurses in the operating room, which is consistent with the results of the present study (33).

Finally, the physical health of the anesthesia workplace was defined by the members as the feeling of comfort and physical security in the operating room. They considered the structure of operating rooms to be the most important cause of fatigue and burnout, and stated that the necessary criteria for the easy travel and settlement of colleagues and their communication with each other were not observed in most surgical places. This seems to be more related to the architectural design of the operating room. Davies et al. stated that the successful performance of anesthesia team members relies on optimization of the ergonomics of the operating room, and if due attention is not paid to these details, their performance will be disrupted. According to their results, temperature, humidity, adequate lighting, and visibility or availability of equipment needed by the anesthesia team member in the operating room are influencing factors in this regard (34). Moreover, amenities are not equally distributed in all operating rooms. Due to the nature of their job, anesthesia personnel have more free time, especially in the evening and night shifts where patient load is less, and adequate rest and relaxation can save them energy to serve possible emergency surgeries in the evening and night shifts.

Our study is worthwhile in that it dealt with a little understood phenomenon and came up with important findings. However, there are a number of limitations that should be addressed. First shortcoming of this study is self-selection bias. The greater likelihood of participation of interviewees with good speaking skills may have affected our results. Given the large number of nurse anesthetists participating in this study, there is a possibility of bias in the findings. In fact, more than half of all participants were nurse anesthetists, and this may have skewed the results toward their attitudes as opposed to those of the anesthesiologists. Of course, more anesthesiologists were supposed to be recruited to alleviate this limitation, but due to their OR cases and schedules, this was not possible.

Despite these limitations, the present study is worthwhile due to the credibility of data analysis. Credibility was enhanced in this study as only one researcher who had no affiliation with the institutions or the participants conducted the interviews. Also taking field notes to capture information and verification of transcript accuracy enhanced the credibility of this study.

## 5. Conclusion

In the present study, interviews were conducted with all members of the anesthesia team, including anesthesiologists, nurse anesthetists, and chief nurse anesthetists, in order to explore the factors affecting workplace health from their perspective. Findings showed that the participants' emphasis is more on behavioral and managerial factors and that desirable interpersonal cooperation in creating a suitable work environment for them is more prominent. These findings can raise the awareness of chief nurse anesthetists and planners to provide more effective teamwork, modify the job description structure, and reduce staff conflicts. Further studies should include senior chief nurse anesthetists, including anesthesiologists and nurse anesthetists.

## Data availability statement

The raw data supporting the conclusions of this article will be made available by the authors, without undue reservation.

## Ethics statement

The studies involving human participants were reviewed and approved by Ahvaz Jundishapur University of Medical Sciences. The patients/participants provided their written informed consent to participate in this study.

## Author contributions

AK: conceptualization, methodology, formal analysis, writing—review and editing, and project administration. NS: conceptualization, methodology, data curation, formal analysis, software, investigation, and writing-original draft. SA: conceptualization, methodology, formal analysis, writing—review and editing, and supervision. All authors contributed to the article and approved the submitted version.
